# Real-World Characteristics and Treatment Patterns of Patients With Transthyretin Amyloid Cardiomyopathy: Protocol for a Multicountry Disease Registry Study

**DOI:** 10.2196/71314

**Published:** 2025-06-06

**Authors:** Yen-Hung Lin, Hsu-Wen Chou, Sarah Tsai, Roy Gomez

**Affiliations:** 1 Division of Cardiology Department of Internal Medicine National Taiwan University Hospital Taipei Taiwan; 2 Cardiovascular Center National Taiwan University Hospital Taipei Taiwan; 3 Medical Affairs Pfizer Ltd Taipei Taiwan; 4 Emerging Markets Asia Specialty Care, Global Medical Affairs Pfizer Private Limited Singapore Singapore

**Keywords:** ATTR-CM, transthyretin amyloid cardiomyopathy, registry, real world data

## Abstract

**Background:**

Transthyretin amyloid cardiomyopathy (ATTR-CM) is a systemic amyloidosis disorder with early clinical manifestations similar to other heart conditions, which complicates its diagnosis and management. The disease’s insidious nature and its progression to heart failure emphasize the critical need for enhanced recognition and understanding of its clinical landscape.

**Objective:**

This study aimed to understand the natural history and current treatment patterns for managing ATTR-CM in a diverse Asian cohort from Taiwan, Hong Kong, and Malaysia.

**Methods:**

This study is a multicenter, noninterventional disease registry that plans to enroll patients diagnosed with ATTR-CM across approximately 17 sites in Taiwan, Hong Kong, and Malaysia. Almost 350 patients with a documented diagnosis of ATTR‑CM after June 1, 2019, will be enrolled in the study. Deceased patients will be enrolled without the need for consent in accordance with applicable regulations. Their data will be gathered retrospectively through a 1-time review of their medical records, where permissible. Data related to clinical characteristics, treatment, and outcomes will be collected for each patient during the routine clinical practice while adhering to local standards of care. The end of data collection is planned for at least 12 months after the end of the enrollment period.

**Results:**

As of March 16, 2025, ethical approvals for this study have been obtained or are under review at multiple sites across Taiwan, Hong Kong, and Malaysia. The study commenced on October 1, 2024, with the first participant’s first visit and so far, 59 patients have been recruited: 35 from National Taiwan University Hospital (Taiwan), 13 from Taipei Veterans General Hospital (Taiwan), 2 from China Medical University Hospital (Taiwan), 2 from Sarawak Heart Center (Malaysia), and 7 from Queen Mary Hospital (Hong Kong). An interim report is scheduled for completion by December 31, 2025. The end of data collection, marked by the last participant’s visit, is planned for October 1, 2027, and the final study report is expected to be finalized by June 1, 2028. Once established, the database will serve as a comprehensive resource for analyzing baseline characteristics, treatment patterns, and outcomes in patients with ATTR-CM from diverse health care systems.

**Conclusions:**

This research will aid in understanding the demographic, clinical, and therapeutic patterns of ATTR-CM in Taiwan, Hong Kong, and Malaysia. This registry may influence advancements in early detection, diagnosis, and tailored treatment strategies in ATTR-CM.

**Trial Registration:**

ClinicalTrials.gov NCT06651073; https://clinicaltrials.gov/study/NCT06651073

**International Registered Report Identifier (IRRID):**

DERR1-10.2196/71314

## Introduction

### Background

Transthyretin amyloid cardiomyopathy (ATTR-CM) is a systemic amyloidosis disorder caused by the accumulation of misfolded transthyretin proteins, leading to the formation of amyloid fibrils in the extracellular space [1]. These fibrils alter tissue structures and impair the function of various organs [1]. Due to nonspecific symptoms, ATTR-CM diagnosis is often delayed or missed [2]. If untreated, ATTR-CM is a fatal disorder, commonly progressing to congestive heart failure (HF), cardiac arrhythmia, conduction system disease, and complete HF [2,3]. Despite its severity, there is a scarcity of research, especially in Asia, on disease progression, treatment practice, clinical outcomes, and disease mortality in ATTR-CM [4].

ATTR‑CM can be classified by the sequence of the *ATTR* gene, either wild-type, with no mutations (wt*ATTR*), or hereditary, with a mutation (h*ATTR*) [5]. Disease progression is generally slower in wt*ATTR*, whereas h*ATTR* often progresses more rapidly due to increased amyloid accumulation [5]. Many different mutations have been found in the *ATTR* gene, leading to variability in the initial signs and symptoms among patients with h*ATTR* depending on their specific genetic mutations [6,7]. In people of Chinese descent in Taiwan and Malaysia, Ala97Ser is found to be the most prevalent genotype, where it accounts for more than 90% of *ATTR* gene variations among affected individuals [8,9].

As ATTR-CM is an underrecognized disease, reported global prevalence rates vary, and the true prevalence remains uncertain [10,11]. A previous study suggested a prevalence rate of approximately 13% in patients with HF and myocardial wall thickening of more than 12 mm [12]. However, there are no large-scale studies, and the actual prevalence of ATTR-CM in Asia remains unclear [4].

Understanding the demographic and clinical characteristics of patients with ATTR-CM across a variety of health care systems is crucial for enhancing the recognition and early detection of the disease [13].

**Figure 1:**
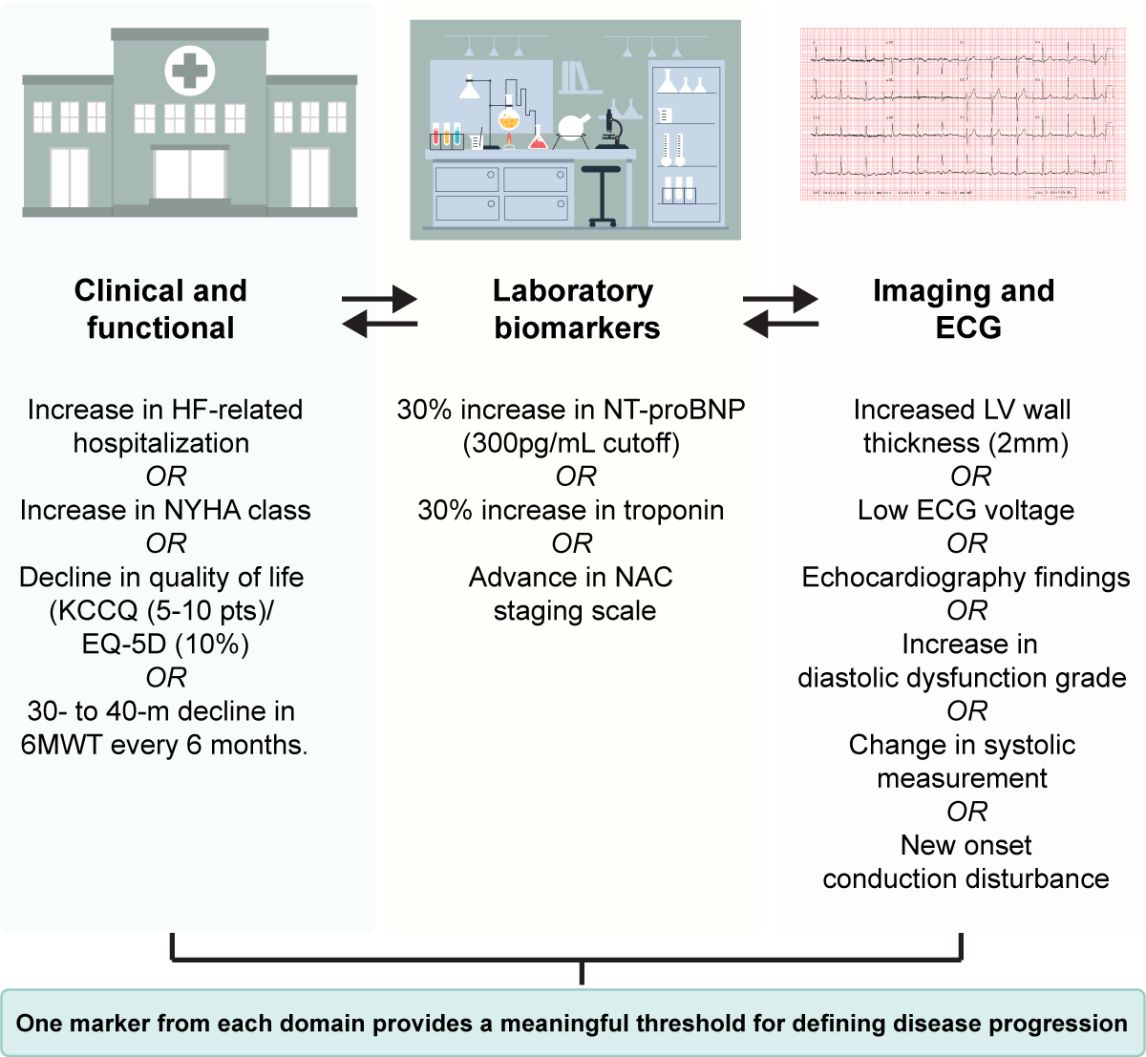
Criteria for assessing disease progression in patients with ATTR-CM. Consensus from experts recommends the three domains above for the long-term monitoring of patients with ATTR-CM. 6MWT: 6-minute walk test; ATTR-CM: transthyretin amyloid cardiomyopathy; ECG: electrocardiography; HF: heart failure; KCCQ: Kansas City Cardiomyopathy Questionnaire; LV: left ventricle; NAC: National Amyloidosis Centre (United Kingdom); NT-proBNP: N-terminal pro-B-type natriuretic peptide; NYHA: New York Heart Association.

A panel of experts from Europe and the United States recommended three key approaches for monitoring the progression of ATTR-CM: (1) clinical and functional endpoints, (2) biomarkers and laboratory markers, and (3) imaging and electrocardiograph parameters (Figure 1) [2,4]. However, across Asian populations, the pathological presentation of ATTR differs, and expert consensus on monitoring the progression of ATTR-CM will vary across regions [14-16]. Due to limitations in health care accessibility across Asia and variations in clinical practice, there are alternative recommendations that take perspectives from Asian countries into consideration (Figure 1) [2,4].

In assessing clinical and functional endpoints, the 6‐minute walk test remains a widely accessible and cost-effective tool in Asia [17]. In addition, measuring cardiac biomarkers, such as serum N‐terminal pro-brain natriuretic peptide and serum cardiac high‐sensitivity troponin, is useful for measuring disease progression [18,19]. However, in the event of poor accessibility, alternatives in imaging and electrocardiogram tools have not yet been established [4]. Clinical experience in Asia suggests, for example, that a “relative” low electrocardiogram voltage as an alternative to left ventricle wall thickness would be an important indicator of ATTR-CM progression in Asian patients [4]. Echocardiography is also suggested, as it is generally available across Asia. Clearly, there is a need for research that augments the methods and tools used to diagnose and monitor ATTR-CM progression. Monitoring these disease domains through a registry would aid in understanding the characteristics and progression of ATTR-CM in Taiwan, Hong Kong, and Malaysia, ultimately, this is needed to improve disease recognition, detection, and appropriate management across these regions [20].

Singapore and Thailand have established country-level patient registries for ATTR-CM; however, there is a pressing need for other Asian countries to develop similar registries as well as compare the data across countries in the Asian region [4,21]. In this article, we describe the protocol for a multicenter, noninterventional disease registry to characterize the natural history of ATTR-CM and treatment patterns in patients clinically diagnosed with ATTR-CM across Taiwan, Hong Kong, and Malaysia. This dataset, spanning 3 diverse nations, is essential for building a robust population base that captures variations in ethnicity, socioeconomic status, and medical insurance coverage, while also reflecting differences in clinical care practices across varied health care systems [13].

### Aims and Objectives

This study aims to understand the natural history and treatment patterns of patients with ATTR-CM in Taiwan, Hong Kong, and Malaysia. Primary, secondary, and exploratory objectives, respectively, are listed in Textbox 1.

**Study objectives.**

- To characterize the natural history of patients with transthyretin amyloid cardiomyopathy (ATTR-CM), described by patient demographic and clinical characteristics at diagnosis of patients with ATTR-CM, and patient outcomes.
- To describe the treatment patterns in patients with ATTR-CM and to identify the prognostic factors for all-cause mortality and cardiovascular-related hospitalization in patients with ATTR-CM.
- To assess treatment effectiveness in patients with ATTR-CM and to evaluate the quality of life and health care resource usage in patients with ATTR-CM.

## Methods

### Study Design and Setting

**Figure 2:**
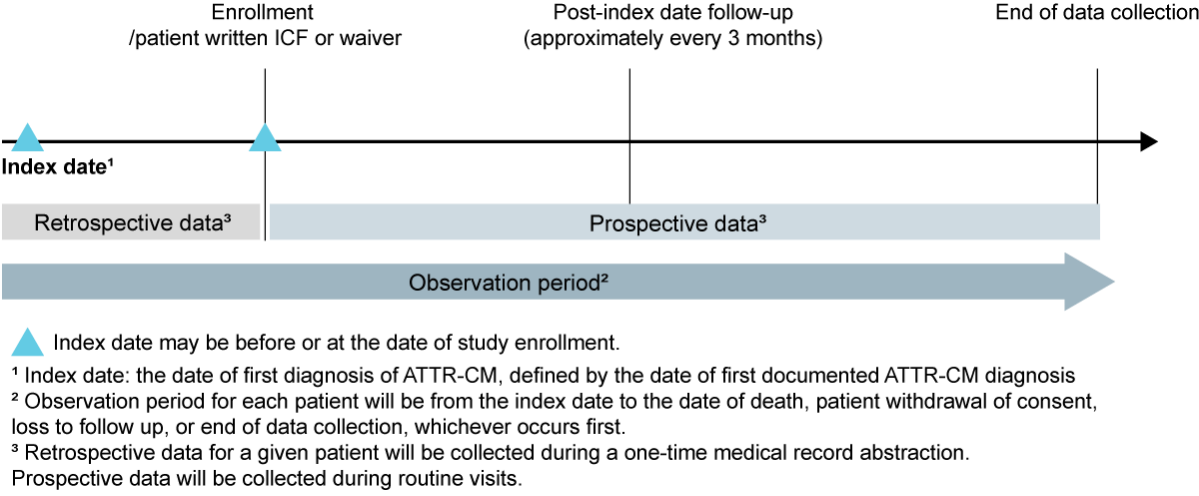
Study design. ATTR-CM: transthyretin amyloid cardiomyopathy; ICF: informed consent form. The “index date” marks the point of first diagnosis, which may coincide with or precede study enrollment. Patients are then followed over time, capturing both retrospective data (before the index date) and prospective data (after the index date), at approximately 3-month intervals. The “observation period” extends from the index date until either the end of the study, patient death, withdrawal of consent, or loss to follow-up. Data are collected through a combination of retrospective review during a one-time medical record abstraction and routine clinical visits for prospective information.

This multicenter, noninterventional, disease registry enrolling approximately 350 patients diagnosed with ATTR-CM will be conducted across approximately 17 sites in Taiwan, Hong Kong, and Malaysia. All patients with a documented diagnosis of ATTR‑CM after June 1, 2019 will be invited to participate in the study. The study size will be based on the number of eligible ATTR-CM cases identified in the medical records and meeting the inclusion criteria. However, the goal is to enroll approximately 350 patients diagnosed with ATTR-CM across the sites, with data collected throughout routine clinical practice. As it is a noninterventional study, no procedures or treatments will be mandated, and patients will receive their usual clinical care. The observation period for each patient will range from the index date (ie, the date of first documented ATTR-CM diagnosis) to whichever occurs first of death, patient withdrawal of consent, loss to follow-up, or end of data collection. The end of data collection is planned for a minimum of 12 months after the end of the enrolment period. This will allow all patients to have the opportunity to contribute at least 12 months of follow-up data (Figure 2).

#### Study Participants

The inclusion criteria of this ATTR-CM registry are as follows: (1) adults (aged ≥18 years) at first ATTR-CM diagnosis; (2) confirmed diagnosis of ATTR-CM after June 1, 2019; and (3) able to provide informed consent. The eligibility criteria are broad enough to include all patients with ATTR-CM without any exclusion criteria.

Several strategies to address potential selection bias will be implemented. Each eligible patient will be consecutively assessed during the designated period using predefined criteria to ensure comprehensive inclusion and reduce bias. Recruitment will span a variety of clinical settings to capture a diverse and representative sample. The eligibility criteria are designed to be broad, promoting inclusivity and enhancing the generalizability of our findings. In addition, a screening log for individuals who do not participate will be maintained to gather minimal deidentified information to identify any systematic differences and further understand potential biases. Each enrolled participant or his or her legally acceptable representative will be provided with an informed consent form (ICF) that outlines the study objectives, procedures, risks, and benefits, and clearly states that participation is voluntary with the option to opt out at any time without affecting their clinical care.

#### Data Collection and Management

All variables described below will be collected as part of routine clinical practice or following standard practice guidelines in the countries where this study is being conducted. This study will not provide or recommend any treatment; all decisions regarding treatment will be made at the sole discretion of the treating physician, in accordance with their routine clinical practice administration of any specific assessment tools is not mandated and all clinical decisions will be made solely at the discretion of the treating physician in accordance with the local and international guidelines. The data to be collected retrospectively at enrollment and prospectively during follow-up is listed in Textbox 2.

**Data to be collected retrospectively at enrollment and prospectively during follow-up.**

- Demographic data (age at enrolment, sex, and race or ethnicity).
- Diagnosis of transthyretin amyloid cardiomyopathy (ATTR-CM; date of first ATTR-CM diagnosis, age at first ATTR-CM diagnosis, method of diagnosis, etc).
- Physical measurements (height, weight, smoking status, blood pressure, and heart rate).
- Relevant medical history and comorbidities (first clinical cardiac symptom related to ATTR-CM, symptom onset date as the date of the first clinical symptom attributed to ATTR-CM, age at onset of symptom, treatment received for the first symptom if applicable with its start and stop dates).
- Emergency department visits.
- Type of ATTR-CM (wild-type ATTR or hereditary ATTR [hATTR] with genetic variants identified if applicable for patients with hATTR).
- Family history (known cardiomyopathy, polyneuropathy, and sudden cardiac death [among parents, siblings, and second- or third-degree family links]).
- Treatment for ATTR-CM (ATTR-specific pharmacologic treatment, including, treatment name, dosage, prescribed frequency, start date, end date, and any change in treatment).
- Prescription of heart and cardiovascular medication (including beta-blockers, angiotensin-converting enzyme inhibitors, angiotensin receptor-neprilysin inhibitors, angiotensin II receptor blockers, digoxin, calcium channel blockers, diuretics, antiplatelets, lipid-lowering agents, and anticoagulants (within 6 months before the index date).
- Concomitant medications (including heart and cardiovascular medications and other concomitant medications from the index date).
- New York Heart Association functional class (closest available measurement to the index date).
- Kansas City Cardiomyopathy Questionnaire.
- Imaging and lab assessments (date of assessment and results).
- Survival status (date of death, reason for death: Cardiovascular-related, not cardiovascular-related, and indeterminate).
- Hospitalization (date of admission, date of discharge, reason for hospitalization categorized as cardiovascular-related, not cardiovascular-related, and indeterminate).
- Adverse events with explicit attribution to any of the study sponsor’s drugs.

Multimedia Appendix 1 provides further details on each data collection element, including the timepoints at which the data will be recorded. The study case report form (CRF) includes an “ATTR-CM diagnosis” section detailing the methods used to diagnose ATTR-CM. The CRF captures a range of clinically validated approaches, including functional classification, imaging, biomarker screening, and genetic testing, consistent with established guidelines for ATTR-CM diagnosis.

The collected data will be from reliable sources of patient-level information available at the participating sites. This includes data available in the patients’ electronic medical records as well as medical charts and any other documentation or communication by health care providers. All data listed above will be collected by trained site personnel and entered directly into the web-based electronic case report forms (eCRFs) at each participating study site. Sites will be responsible for entering extracted patient data into a secure Electronic Data Capture (EDC) database and all changes or corrections to eCRFs will be documented in an audit trail. The personal data will be stored at the study site in encrypted electronic or paper form and will be password-protected or secured in a locked room to ensure that only authorized study staff have access. Patient data will be deidentified before analysis. All identifiable information will be replaced with a unique patient-specific identifier, and data will be transmitted and managed using encryption.

### Data Analysis

Detailed methodology for summary and statistical analyses of data collected in this study is documented in a statistical analysis plan. This study is descriptive and does not involve hypothesis testing, hence the actual sample size will be determined by the number of eligible ATTR-CM cases identified in the medical records that meet the inclusion criteria. Approximately 350 patients diagnosed with ATTR-CM are expected to be enrolled based on feasibility assessments, historical diagnosis rates, and the expected patient volume at the 17 participating sites across Taiwan, Hong Kong, and Malaysia. Continuous variables will be summarized using descriptive statistics, including the number of participants with nonmissing and missing values, mean, SD, median, 25th percentile (Q1), 75th percentile (Q3), minimum, maximum, and median (IQR). Means and medians will be presented with one more decimal place than the raw data, while SDs will be presented with 2 more decimal places. Categorical variables will be summarized using frequency counts (n) and percentages (%), with percentages calculated over nonmissing data.

Time-to-event analysis will be performed using the Kaplan-Meier methodology. Data will be presented as the number of patients at risk, with the event, and censored. The estimated median, Q1, Q3, and the 2-sided 95% CI, using Brookmeyer and Crowley method, will be provided for the main statistical estimators. CIs about a parameter estimate will be presented using the same number of decimal places as the parameter estimate.

### Ethical Considerations

The study will be conducted while adhering to all of the legal and regulatory requirements to ensure the protection of patient personal data. Research practices described in Guidelines for Good Pharmacoepidemiology Practices issued by the International Society of Pharmacoepidemiology, Good Practices for Outcomes Research issued by the International Society for Pharmacoeconomics and Outcomes Research will be followed along with any other applicable national guidelines. There is prospective approval of the study protocol, protocol amendments, waiver of ICF for deceased patients, and other relevant documents (eg, ICFs) from the relevant institutional review boards or independent ethics committees.

Ethics approvals for this study have been obtained or are in progress at multiple sites across Taiwan, Hong Kong, and Malaysia. In Taiwan, approvals have been granted by the Research Ethics Committee for National Taiwan University Hospital (date of approval: August 22, 2024, National Taiwan University Hospital record number 202405071RSB), the Taipei Veterans General Hospital institutional review board (IRB; date of approval June 23, 2024; IRB Taipei Veterans General Hospital number 2024-06-026CC), and the Chang Gung Medical Foundation IRB for both the Linkou and Kaohsiung branches of Chang Gung Memorial Hospital (date of approval September 13, 2024, IRB number 202401282B0). Approvals have also been secured from the IRB of Taichung Veterans General Hospital (date of approval August 22, 2024, number SE24333B), and the Research Ethics Committee of China Medical University & Hospital in Taichung (date of approval September 19, 2024, number: CMUH113-REC3-124). In addition, the application has been approved by the Research Ethics Committee of Taipei Mackay Memorial Hospital (date of approval October 8, 2024, number 24CT039be). Applications are pending approval from the Far Eastern Memorial Hospital and the Chi-Mei Medical Center while an application is being prepared for IRB of Kaohsiung Municipal Siaogang Hospital, which is run by Kaohsiung Medical University.

In Hong Kong, ethics approvals have been obtained from the Hospital Authority’s Central IRB for Queen Elizabeth Hospital and Princess Margaret Hospital (date of approval September 25, 2024, Central IRB reference number CIRB-2024-334-5), the Joint Chinese University of Hong Kong–New Territories East Cluster Clinical Research Ethics Committee (CREC) for Prince of Wales Hospital (date of approval: September 15, 2024, CREC reference number 2024.351) and by the IRB of the University of Hong Kong Hospital Authority Hong Kong West Cluster for Queen Mary Hospital (date of approval January 8, 2025, IRB reference number UW 24-623). An ethics application is being prepared for submission to the Hospital Authority Central IRB for Tuen Mun Hospital. In Malaysia, approvals have been obtained from the Medical Research Ethics Committee of Hospital Sultan Idris Shah in Serdang and the Sarawak Heart Centre (date of approval November 8, 2024, number 24-02846-UQ8). The application is currently under review by the IRB of the Medical Research Ethics Committee of Institut Jantung Negara Sdn Bhd.

Each enrolled participant or his or her legally acceptable representative will be provided with an ICF that outlines the study objectives, procedures, risks, and benefits, and clearly states that participation is voluntary with the option to opt out at any time without affecting their clinical care. No compensation will be provided to study participants.

## Results

The study began on October 1, 2024, with the first participant’s visit, and as of March 16, 2025, a total of 59 patients have been recruited. These include 35 patients enrolled from National Taiwan University Hospital, 13 patients recruited from Taipei Veterans General Hospital, and 2 patients enrolled from China Medical University Hospital in Taiwan. Two patients have been recruited from Sarawak Heart Center in Malaysia, and 7 patients enrolled from Queen Mary Hospital in Hong Kong. We anticipate that data collection will continue robustly, with an interim report scheduled for completion by December 31, 2025. The final phase of data collection, marked by the last participant’s visit, is planned for October 1, 2027, and the final study report is expected to be finalized by June 1, 2028. Once established, the resulting database will serve as a valuable resource for analyzing baseline characteristics, treatment patterns, and outcomes in patients with ATTR-CM. This extensive dataset will not only provide insights into current clinical management but also guide future research efforts and inform evidence-based clinical practices in managing ATTR-CM.

## Discussion

### Anticipated Findings

The aim of this study is to understand the natural history and treatment patterns of patients with ATTR-CM in Taiwan, Hong Kong, and Malaysia. Therefore, a multicenter, noninterventional, disease registry study design was selected. The study aims to recruit patients diagnosed with ATTR-CM from approximately 17 sites in Taiwan, Hong Kong, and Malaysia. To be included in the study, patients will need to be adults (aged ≥18 years) at first ATTR-CM diagnosis or confirmed diagnosis of ATTR-CM after June 1, 2019. Reflected in the small number of global ATTR-CM studies, there is an absence of studies describing the populations of patients with ATTR-CM in Taiwan, Hong Kong, and Malaysia as such this study will ensure that the necessary data is available to improve the understanding of ATTR-CM [4]. The data collection was designed to align with routine clinical practice, following local standard practice guidelines.

ATTR is a variable disease, both phenotypically and geographically and TTR mutations appear to exist on an ethnic-specific spectrum [22-25]. In other studies, the most common TTR mutation is Thr60Ala in the UK and US patients, Val30Met in Japanese patients, Asp38Ala in South Korean patients, and Val30Met in Portuguese, Swedish, and Japanese patients while the Val122Ile mutation is endemic in the African-American population [26]. It is important not to rely solely on global data or data from other countries to inform the manifestation of ATTR-CM in Taiwan, Hong Kong, and Malaysia, particularly as Ala97aSer, the most common endemic mutation across these countries, which is less prevalent across the rest of the world, and as such has fewer reported cases and studies [27,28].

Across the global population as well as within individual nations, ATTR-CM has not received sufficient recognition, diagnosis, or treatment due to low public awareness and the absence of an effective therapeutic approach. However, even though there is a new understanding of the diagnosis, progression, and treatment of this disease worldwide [29], the prevalence and mortality rates based on real-world evidence (RWE) remain unknown in Asia [4]. This gap underscores the necessity for real-world data collection in Asia and this study is an initiative to fill in this knowledge void, starting with a clinical registry across Taiwan, Hong Kong, and Malaysia.

Establishing registries for rare diseases like ATTR-CM is fundamental to enhancing patient care pathways and ultimately improving disease outcomes [30,31]. Such registries are instrumental in generating RWE; they offer a systematic approach to monitor disease progression and assess the efficacy and safety of various treatment modalities [32]. The data compiled as per this protocol will enable a comprehensive understanding of ATTR-CM in the Asian context. An ATTR-CM registry for Asian countries is a critical step toward evaluating health care burden, clinical progression, health care strategies, and potential disparities. It will also facilitate a deeper analysis of treatment efficacy and safety, thereby improving the overall standard of care for patients with ATTR-CM [4].

As a multicenter, noninterventional, disease registry study aiming to collect real-world data on ATTR-CM, there are multiple strengths with this study design. Given the frequent undiagnosed instances of ATTR-CM in various clinical settings, this protocol is designed to ensure that the study sample accurately represents the target population through systematic patient assessment, nonrestrictive recruitment, and inclusive inclusion criteria. However, there are also some potential limitations inherent to the design of any multicountry and multicentric study. Center selection was based on each center’s readiness to participate and the availability of patients diagnosed with ATTR-CM. Consequently, while the protocol uses systematic patient assessment with broad, inclusive criteria, the study sample may not fully capture the national or regional prevalence or diagnosis rates of ATTR-CM. This targeted recruitment approach may introduce selection bias, potentially limiting the generalizability of the findings. Furthermore, variations in routine clinical care and adherence to standard practice guidelines across these multiple sites in different countries may affect the consistency, completeness, and types of data collected.

Despite these limitations, this study aims to make substantial contributions to the limited existing literature on ATTR-CM by documenting the natural history and treatment patterns of patients in Taiwan, Hong Kong, and Malaysia. It holds the potential to improve the detection and management of ATTR-CM in Asia, ultimately improving patient outcomes and increasing survival rates.

### Conclusions

Establishing an ATTR-CM registry across Asian countries is essential for generating real-world evidence on disease burden, clinical progression, health care strategies, and potential disparities, enhancing patient care pathways and informing tailored treatment approaches. By systematically documenting the demographic, clinical, and therapeutic patterns of patients in Taiwan, Hong Kong, and Malaysia, this study will fill critical gaps in the literature on natural history and treatment outcomes for ATTR-CM in the region.

## Appendix

Multimedia Appendix 1 Overview of data collection categories and variables.

## Abbreviations

ATTR-CM: transthyretin amyloid cardiomyopathy
CREC: Clinical Research Ethical Committee
CRF: case report form
eCRF: electronic case report form
EDC: Electronic Data Capture
hATTR: hereditary ATTR
HF: heart failure
ICF: informed consent form
IRB: institutional review board
RWE: real-world evidence
wtATTR: wild-type ATTR
