# Supplementary material for: Real-World Characteristics and Treatment Patterns of Patients With Transthyretin Amyloid Cardiomyopathy: Protocol for a Multicountry Disease Registry Study
Source: JMIR Res Protoc. 2025 Jun 6;14:e71314. doi: 10.2196/71314 (PMC12181751; doi:10.2196/71314)
Supplement: Multimedia Appendix 1 [file resprot_v14i1e71314_app1.docx]

**Overview of data collection categories and variables**

| **Category** | **Variables** | **Collection Method** | **Timepoint(s)** |
| --- | --- | --- | --- |
| **1. Demographics** | - Year of birth - Country of birth - Sex - Ethnicity - Race | - EHR | - At enrollment (retrospective record abstraction at baseline) |
| **2. ATTR-CM Diagnosis** | - Date of first diagnosis - Age at diagnosis - Methods of diagnosis   - New York Heart association class   - Atrial fibrillation   - Pacemaker   - Implantable Cardiac defibrillators   - Cardiac biopsy   - MRI   - ECG   - ECHO   - PYP scan with SPECT/CT (for definitive confirmatory testing)   - pNfL   - NT-proBNP   - eGFR   - Other assessment | - EHR | - Retrospective (index date) |
| **3. Physical Measurements** | - Height - Weight - BMI - Systolic and diastolic blood pressure - Heart rate - Smoking history (Never, Prior, Current, Unknown) | - EHR | - Retrospective (baseline) and prospective (approximately every 3 months) |
| **4. Medical History & Comorbidities** | - Relevant medical history and comorbidities | - EHR | - Retrospective (baseline) and updated prospectively as available |
| **5. Emergency Visits** | - Date of visit - Reason for visit | - EHR | - Prospective (recorded at routine clinical visits, ~every 3 months) |
| **6. ATTR-CM Type** | - Wild type - Hereditary - Unknown | - EHR /Genetic testing | - Retrospective (index date) |
| **7. Family History Including Known Cardiomyopathy, Polyneuropathy, And Sudden Cardiac Death** | - Relationship to patient - Age (years) of diagnosis - Date of diagnosis - Type of disease/event | - EHR | - Retrospective (baseline record abstraction) |
| **8. ATTR-CM Treatment** | - ATTR-CM specific pharmacological treatment   - Treatment name   - Dosage - Supportive treatment for cardiac involvement - Liver or heart transplantation | - EHR | - Prospective (recorded at routine clinical visits, ~every 3 months) |
| **9. Cardiovascular Medications** | - Diuretic - Aldosterone receptor blockers - Beta-blockers - ACE - ARBs - ARNi - Other | - EHR | - Prospective (recorded at routine clinical visits, ~every 3 months) |
| **10. Concomitant Medications** | - Heart-related and other medications since index date | - EHR | - Prospective (recorded at routine clinical visits, ~every 3 months) |
| **11. NYHA Class** | - Functional class (I-IV) | - Clinical assessment | - Retrospective (baseline) and prospective (as available during routine visits) |
| **12. KCCQ Score** | - Kansas City Cardiomyopathy Questionnaire (0-100) | - Clinical assessment | - Retrospective (baseline) and prospective (as available during routine visits) |
| **13. Imaging & Lab Tests** | - Records of imaging and lab assessments | - EHR / Labs | - Retrospective (baseline) and prospective (as available during routine visits) |
| **14. Survival Status** | - Date of death   - Reason for death (CV-related, Non CV-related, Indeterminate) | - EHR | - Prospective (monitored throughout the observation period) |
| **15. Hospitalization** | - Admission/discharge date   - Reason for hospitalisation (CV-related, not CV-related, Indeterminate) | - EHR | - Retrospective (baseline) and prospective (as available during routine visits) |
| **16. Adverse Events** | - Occurrence of adverse events attributed to any medications. - Record and report separately adverse events to medications marketed or in pipeline by clinical trial sponsor | - EHR / Study records | - Prospective (recorded at routine clinical visits, ~every 3 months) |

ARBs = angiotensin II receptor blockers; ARNi = angiotensin receptor–neprilysin inhibitor; ATTR-CM = transthyretin amyloid cardiomyopathy; CV = eardiovascular; ECG = electrocardiogram; ECHO = echocardiogram; EHR = Electronic Health Record; eGFR = estimated glomerular filtration rate; ICD = implantable cardioverter-defibrillator; KCCQ = Kansas City Cardiomyopathy Questionnaire; MRI = magnetic resonance imaging; NT-proBNP = N-terminal pro B-type natriuretic peptide; NYHA = New York Heart Association; pNfL = plasma neurofilament light chain; PYP = Technetium-99m pyrophosphate scan; SPECT/CT = single photon emission computed tomography/computed tomography
